# Bioaccessibility of Maillard Reaction Products from Biscuits Formulated from Buckwheat Flours Fermented by Selected Lactic Acid Bacteria

**DOI:** 10.3390/microorganisms11040883

**Published:** 2023-03-29

**Authors:** Małgorzata Wronkowska, Dorota Szawara-Nowak, Mariusz Konrad Piskuła, Henryk Zieliński

**Affiliations:** Department of Chemistry and Biodynamic of Food, Division of Food Sciences, Institute of Animal Reproduction and Food Research, Polish Academy of Sciences, 10 Tuwima Str., 10-748 Olsztyn, Poland

**Keywords:** fermentation, buckwheat, baking, Maillard reaction products, bioaccessibility

## Abstract

The in vitro bioaccessibility of the soluble protein and Maillard reaction products (MRPs) such as furosine (an early indicator of the MR), free FIC (fluorescent intermediate compounds), and FAST index (fluorescence of advanced MRPs and tryptophan), and the level of melanoidins defined by the browning index were analyzed in biscuits formulated from raw and roasted common buckwheat flours fermented by select lactic acid bacteria (LAB). The content of soluble proteins in fermented buckwheat flour and biscuits before and after digestion in vitro was significantly dependent on the LAB applied and the type of flour used and was the highest in the digested biscuits, indicating increased bioaccessibility. Generally, in all analyzed biscuits a lower furosine content was observed as compared to control samples, and its high bioaccessibility was noted after digestion. The free FIC in biscuits was strain-dependent, resulting in low bioaccessibility with the exception of biscuits obtained from both types of flours fermented by *Streptococcus thermophilus* MK-10. Compared to control biscuits obtained from raw buckwheat flour, the almost twice-increased FAST index was found for samples fermented by *L. plantarum* IB or *Streptococcus thermophilus* MK-10. After digestion, at least a fivefold higher value of the browning index was noted in control and tested biscuits, indicating the high bioaccessibility of melanoidins. This study indicates that fermentation of buckwheat flours by selected lactic acid bacteria seems to be a good way to obtain a product with high bioaccessibility of MRPs. However, further research on their functional properties is needed.

## 1. Introduction

Fermentation is one of the oldest technologies used for the preservation of foods by humans. Native microbiota such as molds, lactic acid bacteria (LAB), enterobacteria, and aerobic spore formers competes for deposits of nutrients in grains of cereal and pseudocereals. Cereals and pseudocereals are good sources of nutrients for many species of the Lactobacillus genus [1,2]. The fermentation process in which LAB is used is above all not very expensive and could improve the overall organoleptic (unique flavor, aroma, or texture) and nutritional characteristics of obtained products. The fermentation of cereal matrix leading to the degradation of antinutritional factors increases the nutritional value of cereal-based food [3].

Buckwheat groats, raw or roasted, are present in the diet of the inhabitants of Central and Eastern Europe. Buckwheat groats are usually served like rice after cooking, while raw buckwheat (groat or flour) is used as a substitute for wheat flour in products for people suffering from celiac disease or gluten sensitivity. The literature data indicate that roasting affects the chemical composition and functional properties of buckwheat groats [4]. Zielińska et al. [5] showed the reduction of antioxidants as well as the formation of Maillard reaction products after buckwheat roasting. Różańska et al. [6] found a significant increase in fluorescent intermediate compounds (FIC) levels in the crumbs and crusts of bread made from roasted buckwheat compared to unroasted ones. The benefits of lactic fermentation of buckwheat were recently described by Zieliński et al. [7] and include the production of organic acids (lactic and acetic), a pH-lowering effect, and the improvement of the shelf life and nutritional properties [8].

Carbonell-Capella et al. [9] presented a definition of bioaccessibility as a fraction of compounds released from the food matrix during the digestion process that can be used for intestinal absorption. These authors presented many factors that affect bioaccessibility, such as the food matrix and its texture and the pH, temperature, and enzymes involved in digestion.

Nooshkam et al. [10] in their review show the pros and cons of the Maillard reaction. These authors show that in food products, MRPs are responsible for a direct aroma and flavor and also improve the antioxidant activity of food. Melanoidins that form during MR are also known for their antimicrobial and antioxidant abilities. It should also be noted that the Maillard reaction led to color loss along with product darkening (at uncontrolled conditions), lysine loss, and the generation of pyridines, furans, and mutagenic compounds as well as advanced glycation end products (AGEs) such as furosine. At present, no information is available on the Maillard reaction products and their bioaccessibility in model buckwheat biscuits. The behavior of some ingredients during the digestive process of buckwheat biscuits prepared from fermented flours using lactic acid bacteria and their bioaccessibility (D-chiro-inositol or quercetin) have been determined and shown by Zieliński et al. [11,12].

Therefore, the present study aimed to determine the bioaccessibility of Maillard reaction products (MRPs) from biscuits formulated from raw and roasted common buckwheat flours after fermentation by select lactic acid bacteria (LAB). The determination of soluble protein and MRPs such as furosine, free FIC (fluorescent intermediate compounds), FAST index (fluorescence of advanced Maillard reaction products and soluble tryptophan), and the level of melanoidins defined by the browning index was addressed.

## 2. Materials and Methods

### 2.1. Materials

Raw buckwheat flour and roasted buckwheat groats originated from Polish commercial common buckwheat (*Fagopyrum esculentum* Moench) purchased from a local industry plant (Melvit S.A., Kruki, Poland). Roasted buckwheat groats were ground in a laboratory mill equipped with screens of different diameters of holes, which resulted in obtaining roasted flour. According to the producer’s declaration, the contents of carbohydrates, dietary fiber, proteins, and fat in buckwheat flour and roasted buckwheat groats were 62 and 69%, 2.3 and 6%, 7.2 and 13%, and 0.7 and 3% of sample dry matter, respectively.

### 2.2. Fermentation of Raw and Roasted Buckwheat Flours, Preparation of Buckwheat Biscuits from Fermented Flours, and In Vitro Digestion

The pretreatment and fermentation process of raw and roasted buckwheat flour and the baking process were conducted according to procedures described in our earlier publication by Wronkowska et al. [13]. Briefly, before the fermentation process about 50 g of each type of flour was suspended with 950 mL of distilled water. Next, the suspension was well stirred during heating at 90 °C for 45 min, then autoclaved at 121 °C/15 min and finally cooled to 37 °C. The pretreatment was carried out to reduce microbial populations existing on buckwheat flours before inoculated fermentation, since they would compete with and inhibit the growth of inoculated microbes during the fermentation process. Then, the 5% suspension of pretreated buckwheat flour in distilled water was inoculated with lactic acid bacteria at the level of 10^8^ CFU/mL, and the fermentation was carried out at 37 °C for 24 h. The following 14 selected lactic acid bacteria strains were used for fermentation: *Lactobacillus acidophilus* (145, La5, V); *Lacticaseibacillus casei*, formerly *Lactobacillus casei* (LcY, 2K); *Lactobacillus delbrueckii* subsp. bulgaricus (151, K); *Lactiplantibacillus plantarum,* formerly *Lactobacillus plantarum* (W42, IB); *Lacticaseibacillus rhamnosus,* formerly *Lactobacillus rhamnosus* (GG, 8/4, K); *Ligilactobacillus salivarius*, formerly *Lactobacillus salivarius* AWH; and *Strepcococcus thermophilus* Mk-10. All of these strains originated from the Institute of Animal Reproduction and Food Research of the Polish Academy of Sciences’ collections. After fermentation, the samples were freeze-dried (Christ–Epsilon 2-6D LSC plus, Osterode am Harz, Germany). Biscuits were baked at 220 °C for 30 min (electric oven DC-21 model, Sveba Dahlen AB, Fristad, Sweden). The control biscuits were formulated on nonfermented buckwheat flour (raw or roasted). The buckwheat biscuits were lyophilized, milled, and stored in a refrigerator until analysis.

The procedure described by Zieliński et al. [7] for in vitro digestion was used in this study. Briefly, lyophilized and milled buckwheat biscuits were suspended in deionized water, and then an α-amylase solution was added to the samples. Samples were shaken in a water bath at 37 °C for 30 min. For gastric digestion, the pH was reduced to 2.0, a pepsin solution was added, and the incubation was continued under the same conditions for 120 min. In the next step, the pH was adjusted to 6.0, and a mixture of pancreatin and bile salts extract was added. Subsequently, the pH was increased to 7.5, and the samples were incubated at 37 °C for 120 min. After incubation, the digestive enzymes were inactivated by heating at 100 °C for 4 min and cooled for centrifugation, and obtained supernatants were stored at −18 °C.

### 2.3. Determination of Maillard Reaction Products and Their Bioaccessibility

Analysis of soluble proteins in all analyzed samples was done according to Wronkowska et al. [13]. Briefly, aliquots of 25 μL of blank, standard, or appropriately diluted samples were added to 200 μL of a bicinchoninic acid BCA solution (BCA: CuSO_4_·5H_2_O, 50:1, *v*/*v*). The mixture was incubated at 37 °C for 30 min, and absorbance was read at λ = 562 nm. The standard curve was plotted within the range of 0.03–1.0 mg/mL bovine serum albumin.

Assays of furosine, free FIC (fluorescent intermediate compounds), FAST index (fluorescence of advanced Maillard reaction products and soluble tryptophan), and browning index were conducted according to methods described in detail by Michalska et al. [14]. Briefly, for the analysis of furosine, FIC, FAST index, and browning index dry samples were mixed with 6% of aqueous SDS (sodium dodecyl sulfate), incubated for 30 min with stirring every 10 min for 30 s, and filtered, and then the filtrates were used for the analysis. Furosine (2-furoylmethyl-lysine) content was evaluated using the chromatographic method. The samples were hydrolyzed with 8 mL of 8 M HCl at 110 °C for 23 h under anaerobic conditions, and then the hydrolysates were filtered and used for further analysis. Quantitative analysis of furosine was performed based on the external standard method using a commercial standard of pure furosine. The free fluorescent intermediate compounds (FIC) were measured at λEx = 353 and λEm = 438 nm. Measurement of fluorescent advanced Maillard reaction products (MRPs) and calculation of the FAST index were based on the analysis of fluorescence due to advanced MRPs measured at λEx = 353 and λEm = 438 nm and tryptophan fluorescence measured at λEx = 290 and λEm = 340 nm. The formation of brown pigments in the examined buckwheat samples was estimated as absorbance at 420 nm.

To determine the in vitro bioaccessibility index (BI), the content of analyzed Maillard reaction products after digestion was divided by the content of the product before digestion.

### 2.4. Statistical Analysis

The measurements were performed in at least 3 replications for each type of buckwheat biscuits obtained from 3 separate baking processes for every formulation. The reported data are the mean results for each formulation with the standard deviation. To determine the difference resulting from the fermentation process used as compared to the control sample, Student’s *t*-test was used. One-way ANOVA (*p* < 0.05) was used to determine the difference resulting also from the baking and digestion processes used. The Fisher LSD Test, at a significance level of *p* < 0.05, was performed for post hoc comparison. All analyses were made using STATISTICA for Windows (StatSoft Inc., Tulsa, OK, USA, 2001).

## 3. Results

**Figure 1:**
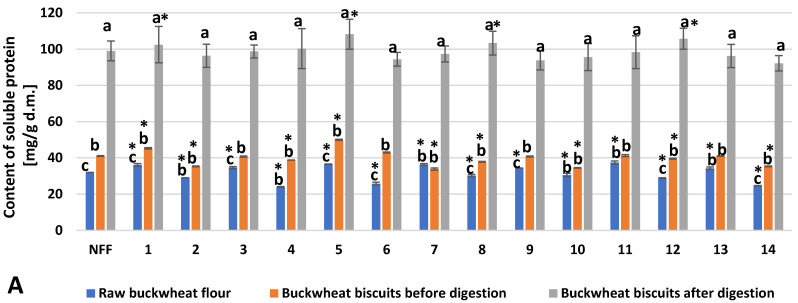

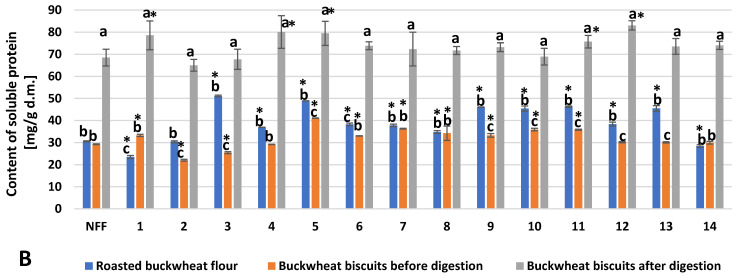
Content of soluble protein in raw (**A**) and roasted (**B**) buckwheat flour fermented with selected strains of lactic acid bacteria and in buckwheat biscuits before and after in vitro digestion (mg/g d.m.). Data are expressed as mean ± standard deviation (*n* = 3). * Upper star means the significant differences at *p* < 0.05 of fermented samples as compared to the control sample based on Student’s *t*-test. A letter means significant differences at *p* < 0.05 based on one-way ANOVA. NFF—nonfermented flour. Flours fermented by: 1*—Lactiplantibacillus plantarum* IB; 2—*Lactiplantibacillus plantarum* W42; 3—*Lactobacillus delbrucki* subsp. *bulgaricus* 151; 4—*Lacticaseibacillus casei* Lcy; 5—*Streptococcus thermophilus* MK-10; 6—*Lactobacillus acidophilus* La5; 7—*Lactobacillus acidophilus* V; 8—*Lactobacillus acidophilus* 145; 9—*Lacticaseibacillus casei* 2K; 10—*Lactobacillus delbrucki* subsp. *bulgaricus* K; 11—*Lacticaseibacillus rhamnosus* GG; 12—*Lacticaseibacillus rhamnosus* 8/4; 13—*Lacticaseibacillus rhamnosus* K; 14—*Ligilactobacillus salivarius* AWH.

**Figure 2:**
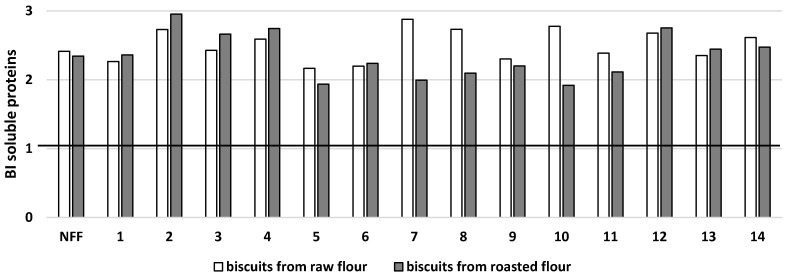
Potential bioaccessibility of soluble protein (BI soluble proteins) from buckwheat biscuits baked from raw or roasted buckwheat flour fermented by selected strains of lactic acid bacteria. NFF—nonfermented flour. Flours fermented by: 1*—Lactiplantibacillus plantarum* IB; 2—*Lactiplantibacillus plantarum* W42; 3—*Lactobacillus delbrucki* subsp. *bulgaricus* 151; 4—*Lacticaseibacillus casei* Lcy; 5—*Streptococcus thermophilus* MK-10; 6—*Lactobacillus acidophilus* La5; 7—*Lactobacillus acidophilus* V; 8—*Lactobacillus acidophilus* 145; 9—*Lacticaseibacillus casei* 2K; 10—*Lactobacillus delbrucki* subsp. *bulgaricus* K; 11—*Lacticaseibacillus rhamnosus* GG; 12—*Lacticaseibacillus rhamnosus* 8/4; 13—*Lacticaseibacillus rhamnosus* K; 14—*Ligilactobacillus salivarius* AWH. BI > 1: high bioaccessibility; BI < 1: low bioaccessibility.

Soluble protein was present in fermented raw buckwheat flours in the range of 23.9–36.5 mg/g d.m. vs. 31.9 mg/g d.m. (unfermented raw buckwheat flour) (Figure 1A). In turn, in fermented roasted buckwheat flours, soluble protein was found in the range of 23.5–51.1 mg/g d.m. vs. 30.5 mg/g d.m. as determined for unfermented roasted buckwheat flour (Figure 1B). Compared to control biscuits obtained from raw buckwheat flour, the significantly higher content of soluble protein in biscuits baked from fermented flour was found only for two samples (Figure 1A), whereas for seven samples in the case of biscuits obtained from fermented roasted buckwheat flour, the increase was about 15–40% (Figure 1B). Digestion of biscuits baked from both types of fermented buckwheat flour led to a 2–3-fold increase in the level of soluble protein compared to its content in the biscuits before digestion (Figure 1A,B). It could therefore be concluded that the digestion of buckwheat biscuits baked from both types of fermented flours leads to an increase in the bioaccessibility of soluble protein, as shown in Figure 2. The same increase in bioaccessibility was also found for control samples baked from nonfermented flours.

The profile of Maillard reaction products in biscuits baked from unfermented and fermented raw and roasted buckwheat flours included the level of furosine formed in the early phase of the Maillard reaction [15,16], the fluorescence of advanced intermediates, tryptophan fluorescence, and the so-called the FAST index [16,17] and the level of melanoidins determined by the browning index [16].

**Figure 3:**
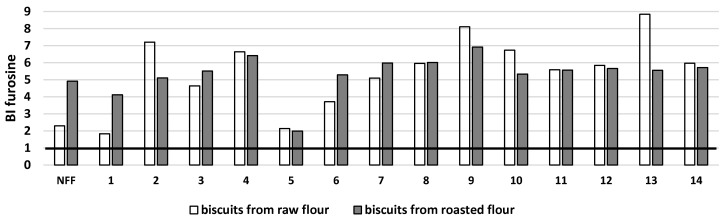
Potential bioaccessibility of furosine (BI furosine) from buckwheat biscuits baked from raw or roasted buckwheat flour fermented by selected strains of lactic acid bacteria. NFF—nonfermented flour. Flours fermented by: 1*—Lactiplantibacillus plantarum* IB; 2—*Lactiplantibacillus plantarum* W42; 3—*Lactobacillus delbrucki* subsp. *bulgaricus* 151; 4—*Lacticaseibacillus casei* Lcy; 5—*Streptococcus thermophilus* MK-10; 6—*Lactobacillus acidophilus* La5; 7—*Lactobacillus acidophilus* V; 8—*Lactobacillus acidophilus* 145; 9—*Lacticaseibacillus casei* 2K; 10—*Lactobacillus delbrucki* subsp. *bulgaricus* K; 11—*Lacticaseibacillus rhamnosus* GG; 12—*Lacticaseibacillus rhamnosus* 8/4; 13—*Lacticaseibacillus rhamnosus* K; 14—*Ligilactobacillus salivarius* AWH. BI > 1: high bioaccessibility; BI < 1: low bioaccessibility.

Furosine was measured as an early indicator of the Maillard reaction (Table 1A,B). It should be noted that raw buckwheat flours, both unfermented and fermented, did not contain furosine, while those from roasted buckwheat, due to the heat treatment used, contained no more than 0.75 mg/g d.m. The presence of furosine in biscuits baked from both types of fermented buckwheat flour was observed. Compared to control buckwheat biscuits from raw and roasted flour, a decrease of furosine content in biscuits, except for a 2-fold increase noted for biscuits obtained from flours fermented by *L. plantarum* IB and *Streptococcus thermophilus* MK-10, was found. The digestion process led to a 2–8-fold increase in the level of furosine compared to nondigested biscuits. It can therefore be concluded that the digestion of buckwheat biscuits baked from fermented flours led to an increase in the bioaccessibility of furosine, as shown in Figure 3. Furosine, which is directly derived from Amadori products, has been considered a useful indicator of the degree of thermal damage during the initial stage of the Maillard reaction in cereal products and is an indirect measure of the available lysine in the food as was presented by Guerra-Hernández et al. [18]. Mesías et al. [19] showed the data connected with the furosine content in commercial breakfast cereals available in the Spanish market and monitored their evaluation from 2006 to 2018 in terms of thermal damage. The authors found about a 40% decrease in furosine content compared to data collected in 2006. They associate this decrease with changes that have been introduced in the last decade in the manufacturing process, the type of grain used, and the presence of honey. Yıltırak et al. [20] found that heating yeast and sourdough fermented native and sprouted wholemeal did not lower the furosine content, but sourdough fermentation unexpectedly increased its content.

**Figure 4:**
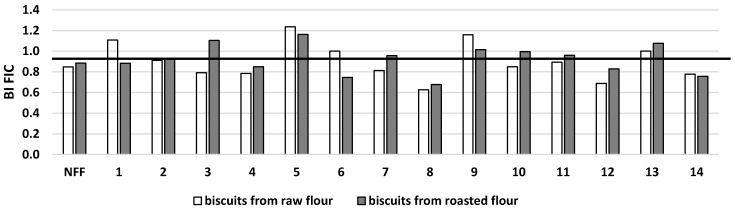
Potential bioaccessibility of free FIC (BI FIC) from buckwheat biscuits baked from raw or roasted buckwheat flour fermented by selected strains of lactic acid bacteria. NFF—nonfermented flour. Flours fermented by: 1*—Lactiplantibacillus plantarum* IB; 2—*Lactiplantibacillus plantarum* W42; 3—*Lactobacillus delbrucki* subsp. *bulgaricus* 151; 4—*Lacticaseibacillus casei* Lcy; 5—*Streptococcus thermophilus* MK-10; 6—*Lactobacillus acidophilus* La5; 7—*Lactobacillus acidophilus* V; 8—*Lactobacillus acidophilus* 145; 9—*Lacticaseibacillus casei* 2K; 10—*Lactobacillus delbrucki* subsp. *bulgaricus* K; 11—*Lacticaseibacillus rhamnosus* GG; 12—*Lacticaseibacillus rhamnosus* 8/4; 13—*Lacticaseibacillus rhamnosus* K; 14—*Ligilactobacillus salivarius* AWH. BI > 1: high bioaccessibility; BI < 1: low bioaccessibility.

The fluorescence of advanced intermediate products (free FIC) from biscuits baked from fermented raw buckwheat flour was in the range of 59.0–107.7 FI/mg d.m. vs. 78.9 FI/mg d.m. for biscuits baked from nonfermented raw flour (Table 1A). The free FIC from biscuits obtained from fermented roasted buckwheat flour was in the lower range of 49.3–76.0 FI/mg d.m. vs. 55.9 FI/mg d.m. found for the control sample obtained from nonfermented roasted buckwheat flour (Table 1B). In vitro digestion of biscuits indicated that the free FIC of the Maillard reaction was strain-dependent (Table 1A,B). Generally, the bioaccessibility of free FIC from analyzed biscuits was lower than one (Figure 4), although for samples obtained from both types of flours fermented by *Streptococcus thermophilus* MK-10, the BI FIC was higher than one.

The analysis of fluorescence associated with the presence of tryptophan in the buckwheat biscuits (Table 1A,B) indicated a slight variation compared to the control biscuits. However, in vitro digestion of the biscuits resulted in the significant release of tryptophan, as the fluorescence level was 3–4 times higher than that found for the biscuits before digestion (Table 1A,B).

Based on analysis of the products of the advanced stage of the Maillard reaction, it was possible to calculate the nutritional value of model buckwheat biscuits in the form of the so-called FAST index (fluorescent of advanced Maillard reaction products and soluble tryptophan). The calculated values of the FAST index for buckwheat biscuits baked from fermented raw flour ranged from 215.3 to 538.7% compared to 262.2% characterizing biscuits baked from unfermented raw flour (Table 1A). Generally, for buckwheat biscuits baked from fermented roasted flour, the values of the FAST index were similar for raw flour (Table 1B). Birlouez-Aragon et al. [21] proposed a rapid fluorimetric method determination of the FAST index to estimate the heat treatment of liquid milk. The FAST index is a sensitive indicator, providing reliable information on the nutritional damage induced by heat treatment [22]. The lower value of the FAST index is an indicator of the higher nutritional value of the products, and it was possible to determine the differentiated effect of fermented buckwheat flour on the nutritional value of biscuits. In this context, the lowest nutritional value both before and after in vitro digestion was found in biscuits baked from buckwheat flour fermented by *L. plantarum* IB or *Streptococcus thermophilus* MK-10 (Table 1A,B). Analogous relationships were found for buckwheat biscuits baked from fermented roasted flour. It should also be noted that the use of fermented buckwheat flour for baking did not increase the nutritional value compared to control biscuits in the context of the FAST index analysis. The observed 3–4-fold decrease in the FAST index after digestion, in the absence of changes in free FIC, was rather a consequence of the increase in the level of tryptophan after digestion.

The marker of the final stage of the Maillard reaction is the so-called browning index, closely related to the level of high-molecular melanoidin. The beneficial presence of these polymeric compounds in food is related to their influence on the sensory properties of food. The browning index for buckwheat biscuits obtained from raw fermented flour was in the range of 0.21–1.05 AU compared to 0.21 AU for biscuits baked from nonfermented flour. The significantly highest values were found for samples obtained from flour fermented by *L. salivarius* AWH, *L. plantarum* IB, and *L. casei* Lcy. The remaining buckwheat biscuits did not differ from the value recorded for the control sample (Table 1A). On the other hand, for biscuits baked from fermented roasted buckwheat flour, the browning index was in a narrower range of 0.30–0.53 AU compared to 0.31 AU for the control sample. Values almost two times higher were observed for biscuits obtained from roasted flour fermented by *L. casei* Lcy and *L. salivarius* AWH compared to the control sample (Table 1B).

**Figure 5:**
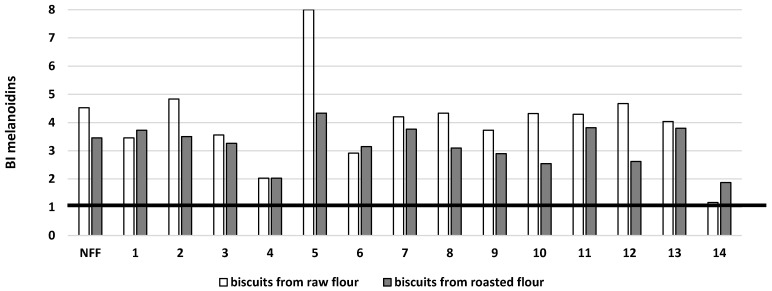
Potential bioaccessibility of melanoidin (BI melanoidin) from buckwheat biscuits baked from raw or roasted buckwheat flour fermented by selected strains of lactic acid bacteria. NFF—nonfermented flour. Flours fermented by: 1*—Lactiplantibacillus plantarum* IB; 2—*Lactiplantibacillus plantarum* W42; 3—*Lactobacillus delbrucki* subsp. *bulgaricus* 151; 4—*Lacticaseibacillus casei* Lcy; 5—*Streptococcus thermophilus* MK-10; 6—*Lactobacillus acidophilus* La5; 7—*Lactobacillus acidophilus* V; 8—*Lactobacillus acidophilus* 145; 9—*Lacticaseibacillus casei* 2K; 10—*Lactobacillus delbrucki* subsp. *bulgaricus* K; 11—*Lacticaseibacillus rhamnosus* GG; 12—*Lacticaseibacillus rhamnosus* 8/4; 13—*Lacticaseibacillus rhamnosus* K; 14—*Ligilactobacillus salivarius* AWH. BI > 1: high bioaccessibility; BI < 1: low bioaccessibility.

Digestion of buckwheat biscuits prepared from fermented raw or roasted flour led to an increase in the bioaccessibility of melanoidin (BI Melanoidins > 1) because the browning index was several times higher after digestion. It should be noted, however, that the browning index was also several times higher after the digestion of both types of control samples, prepared from nonfermented flours. The bioaccessibility of melanoidin for buckwheat biscuits baked from fermented raw flour compared to biscuits from roasted flour was at a comparable level (Figure 5). In only the sample obtained from raw flour fermented by *Streptococcus thermophilus* MK-10, the bioaccessibility of melanoidin was much higher compared to other samples. Melanoidins have attracted much attention as not only a functional food ingredient but also as a potentially healthy dietary supplement [23]. As was presented by Brudzynski and Miotto [24], the formation of melanoidins in the Maillard reaction is a key mechanism underlying honey’s antibacterial and antioxidant activities.

## 4. Conclusions

In this study, the bioaccessibility of Maillard reaction products (MRPs) from biscuits formulated from raw and roasted buckwheat flours originating from common buckwheat after fermentation by select lactic acid bacteria was studied. The soluble protein and Maillard reaction products such as furosine, free FIC (fluorescent intermediate compounds), and FAST index (fluorescence of advanced Maillard reaction products and soluble tryptophan) and the level of melanoidins defined by the browning index were analyzed in the biscuits prepared from fermented flours as compared to the control biscuits prepared from nonfermented ones. The content of soluble proteins in fermented buckwheat flours in prepared biscuits before and after digestion in vitro was significantly dependent on the lactic acid bacteria applied and the type of flour and was highest in the digested biscuits, indicating increased bioaccessibility. Furosine, as an early indicator of the Maillard reaction, showed high bioaccessibility. Generally, the fermentation process used did not influence furosine content. Only for biscuits obtained from flour fermented with *Streptococcus thermophilus* MK-10 was the content of furosine higher compared to biscuits obtained from nonfermented flours. The free FIC in biscuits was strain-dependent, resulting in low bioaccessibility with the exception of biscuits obtained from both types of flours fermented by *Streptococcus thermophilus* MK-10. The fermented buckwheat flours, especially those fermented with *L. plantarum* IB or *Streptococcus thermophilus* MK-10, decreased the nutritional value of biscuits in comparison to the control biscuits in the context of the twice-increased FAST index. The twice-higher browning index was found in biscuits obtained from raw flours fermented by *L. salivarius* AWH and *L. plantarum* IB compared to control samples. At least a fivefold higher value of the browning index was noted in control and tested biscuits after digestion in vitro, indicating a high bioaccessibility of melanoidins. However, further research on the functional properties of baking products obtained from fermented buckwheat flour is needed.

## Figures and Tables

**Table 1:** (**A**). Maillard reaction products in biscuits from raw buckwheat flour fermented with selected strains of lactic acid bacteria before and after in vitro digestion. (**B**). Maillard reaction products in biscuits from roasted buckwheat flour fermented with selected strains of lactic acid bacteria before and after in vitro digestion.

**(A)**										
**Strain/Sample**	**Furosine** **(mg/g d.m.)**		**Free FIC** **(FI/mg d.m.)**		**Tryptophan** **(FI/mg d.m.)**		**FAST Index** **(%)**		**Browning Index** **(AU)**	
	**Before**	**After**	**Before**	**After**	**Before**	**After**	**Before**	**After**	**Before**	**After**
Nonfermented raw flour	3.28 ± 0.04 b	7.53 ± 0.51 a	78.9 ± 3.9 a	66.9 ± 0.8 b	30.1 ± 0.9 b	120.1 ± 3.4 a	262.2 ± 18.4 a	55.7 ± 2.1 b	0.21 ± 0.01 b	0.96 ± 0.02 a
Raw flour fermented by:										
*Lactiplantibacillus plantarum* IB	6.58 ± 0.05 *b	12.05 ± 0.79 *a	69.0 ± 3.0 *a	76.4 ± 6.0 * a	17.7 ± 5.3 *b	65.5 ± 23.7 *a	471.9 ± 27.4 *a	116.7 ± 9.4 *b	0.63 ± 0.25 *b	2.18 ± 0.79 *a
*Lactiplantibacillus plantarum* W42	0.72 ± 0.03 *b	5.16 ± 0.51 *a	67.3 ± 3.0 *a	61.3 ± 1.9 b	31.2 ± 1.8 b	124.5 ± 3.7 a	215.3 ± 18.6 a	49.2 ± 1.6 b	0.22 ± 0.01 b	1.06 ± 0.36 a
*Lactobacillus delbrucki* subsp. *bulgaricus* 151	0.94 ± 0.02 *b	4.38 ± 0.40 *a	96.4 ± 0.5 *a	76.3 ± 1.2 * b	33.1 ± 1.3 b	119.4 ± 4.8 a	291.7 ± 11.5 a	63.9 ± 3.4 b	0.23 ± 0.01 b	0.84 ± 0.10 a
*Lacticaseibacillus casei* Lcy	0.79 ± 0.02 *b	5.25 ± 0.47 *a	80.9 ± 3.0 a	63.4 ± 0.1 b	32.1 ± 0.7 b	123.4 ± 2.7 a	251.8 ± 8.3 a	51.4 ± 1.1 b	0.47 ± 0.01 *b	0.96 ± 0.05 a
*Streptococcus thermophilus* MK-10	6.64 ± 0.01 *b	14.20 ± 1.29 *a	79.8 ± 0.7 b	98.6 ± 2.3 *a	14.8 ± 0.6 *b	74.9 ± 5.3 *a	538.7 ± 22.2 *a	131.6 ± 12.5 *b	0.21 ± 0.02 b	1.74 ± 0.08 *a
*Lactobacillus acidophilus* La5	0.99 ± 0.02 *b	3.67 ± 0.17 *a	68.7 ± 2.8 *a	68.7 ± 2.0 a	30.5 ± 2.4 b	115.4 ± 2.8 a	225.3 ± 8.8 a	59.5 ± 2.4 b	0.23 ± 0.01 b	0.66 ± 0.08 *a
*Lactobacillus acidophilus* V	1.08 ± 0.02 *b	5.50 ± 0.66 *a	94.9 ± 1.3 *a	77.1 ± 4.2 *b	33.7 ± 1.3 b	126.2 ± 2.9 a	281.9 ± 15.1 a	61.1 ± 2.5 b	0.22 ± 0.02 b	0.93 ± 0.07 a
*Lactobacillus acidophilus* 145	0.93 ± 0.02 *b	5.54 ± 0.50 *a	105.5 ± 0.5 *a	66.1 ± 8.8 b	39.6 ± 1.6 *b	119.5 ± 16.2 a	266.2 ± 10.0 a	55.3 ± 1.2 b	0.23 ± 0.01 b	1.00 ± 0.17 a
*Lacticaseibacillus casei* 2K	0.71 ± 0.01 *b	5.74 ± 0.12 * a	59.0 ± 1.6 *b	68.4 ± 2.1 a	25.1 ± 0.9 *b	129.6 ± 6.2 a	234.7 ± 11.5 a	52.8 ± 2.1 b	0.26 ± 0.01 b	0.96 ± 0.13 a
*Lactobacillus delbrucki* subsp. *bulgaricus* K	0.89 ± 0.02 *b	6.00 ± 0.13 a	69.6 ± 1.8 *a	59.1 ± 9.2 * a	26.8 ± 0.0 b	104.0 ± 12.7 *a	259.5 ± 6.4 a	56.8 ± 2.1 b	0.23 ± 0.01 b	0.99 ± 0.05 a
*Lacticaseibacillus rhamnosus* GG	1.13 ± 0.02 *b	6.30 ± 0.25 a	61.8 ± 0.8 *a	55.2 ± 7.5 * a	24.1 ± 1.3 *b	103.5 ± 15.1 *a	256.5 ± 16.9 a	53.4 ± 8.3 b	0.21 ± 0.01 b	0.92 ± 0.05 a
*Lacticaseibacillus rhamnosus* 8/4	0.98 ± 0.02 *b	5.76 ± 0.26 * a	107.7 ± 3.9 *a	74.1 ± 2.1 * b	35.7 ± 2.7 *b	119.0 ± 6.0 a	301.2 ± 23.3 *a	62.2 ± 1.8 b	0.24 ± 0.02 b	1.12 ± 0.17 a
*Lacticaseibacillus rhamnosus* K	0.74 ± 0.02 *b	6.50 ± 0.31 a	67.8 ± 0.6 *a	67.8 ± 4.8 a	28.3 ± 0.7 b	125.4 ± 8.9 a	239.5 ± 7.9 a	54.1 ± 3.0 b	0.21 ± 0.01 b	0.85 ± 0.06 a
*Ligilactobacillus salivarius* AWH	0.60 ± 0.02 *b	3.58 ± 0.20 * a	88.7 ± 2.3 *a	68.9 ± 2.3 b	31.4 ± 1.7 *b	109.1 ± 10.9 a	282.8 ± 22.8 a	63.1 ± 5.1 b	1.05 ± 0.02 *b	1.23 ± 0.09 a
**(B)**										
**Strain/sample**	**Furosine** **(mg/g d.m.)**		**Free FIC** **(FI/mg d.m.)**		**Tryptophan** **(FI/mg d.m.)**		**FAST Index** **(%)**		**Browning Index** **(AU)**	
	**Before**	**After**	**Before**	**After**	**Before**	**After**	**Before**	**After**	**Before**	**After**
Nonfermented roasted flour	1.06 ± 0.04 b	5.20 ± 0.83 a	55.9 ± 0.6 a	49.4 ± 1.6 b	26.1 ± 0.7 b	89.8 ± 3.7 a	214.0 ± 4.6 a	55.0 ± 0.5 b	0.32 ± 0.01 b	1.09 ± 0.04 a
Roasted flour fermented by:										
*Lactiplantibacillus plantarum* IB	1.00 ± 0.03 b	4.13 ± 0.23 a	60.9 ± 1.7 *a	53.8 ± 2.5 b	29.6 ± 0.5 *b	86.6 ± 2.4 a	205.7 ± 7.7 a	62.1 ± 1.3 b	0.31 ± 0.02 b	1.17 ± 0.15 a
*Lactiplantibacillus plantarum* W42	0.96 ± 0.01 *b	4.91 ± 0.28 a	61.0 ± 1.1 *a	56.9 ± 1.9 *b	27.8 ± 1.4 b	91.9 ± 0.6 a	219.7 ± 13.3 a	61.9 ± 2.4 b	0.30 ± 0.03 b	1.05 ± 0.03 a
*Lactobacillus delbrucki* subsp. *bulgaricus* 151	0.91 ± 0.02 *b	5.03 ± 0.78 a	51.2 ± 2.8 a	56.5 ± 2.0 *a	21.2 ± 0.3 *b	86.9 ± 1.4 a	241.5 ± 12.4 *a	65.1 ± 3.1 *b	0.31 ± 0.02 b	1.00 ± 0.10 a
*Lacticaseibacillus casei* Lcy	0.95 ± 0.04 *b	6.07 ± 0.20 a	68.2 ± 1.8 *a	57.9 ± 0.9 *b	30.6 ± 0.5 *b	89.3 ± 1.3 a	222.7 ± 2.9 a	64.9 ± 1.7 *b	0.53 ± 0.03 *b	1.07 ± 0.06 a
*Streptococcus thermophilus* MK-10	6.16 ± 0.04 *b	12.26 ± 0.83 *a	59.4 ± 3.0 b	69.0 ± 1.2 *a	17.3 ± 1.9 *b	79.0 ± 6.3 a	349.9 ± 26.2 *a	88.1 ± 9.0 *b	0.29 ± 0.03 b	1.24 ± 0.11 a
*Lactobacillus acidophilus* La5	1.07 ± 0.02 b	5.66 ± 0.13 a	72.5 ± 1.8 *a	54.1 ± 3.2 b	31.1 ± 1.5 *b	85.3 ± 4.7 a	234.2 ± 15.0 a	63.4 ± 0.9 *b	0.32 ± 0.01 b	1.00 ± 0.03 a
*Lactobacillus acidophilus* V	0.96 ± 0.03 *b	5.74 ± 0.05 a	57.9 ± 0.4 a	55.4 ± 0.8 b	23.6 ± 0.8 *b	90.9 ± 1.0 a	245.1 ± 6.5 *a	61.0 ± 1.0 b	0.29 ± 0.03 b	1.08 ± 0.04 a
*Lactobacillus acidophilus* 145	0.94 ± 0.01 *b	5.66 ± 0.43 a	76.0 ± 1.6 *a	51.4 ± 2.1 b	29.1 ± 1.1 *b	85.5 ± 4.1 a	261.7 ± 10.1 *a	60.2 ± 2.1 b	0.34 ± 0.02 b	1.04 ± 0.04 a
*Lacticaseibacillus casei* 2K	0.77 ± 0.01 *b	5.35 ± 0.26 a	52.0 ± 0.6 a	52.8 ± 3.4 a	22.0 ± 0.6 *b	91.5 ± 6.4 a	236.3 ± 3.9 a	57.6 ± 0.4 b	0.34 ± 0.01 b	0.99 ± 0.11 a
*Lactobacillus delbrucki* subsp. *bulgaricus* K	0.97 ± 0.01 *b	5.16 ± 0.26 a	55.8 ± 1.7 a	55.5 ± 1.6 a	24.7 ± 0.7 b	89.7 ± 3.5 a	225.7 ± 3.7 a	61.8 ± 1.3 b	0.39 ± 0.01 *b	0.98 ± 0.05 a
*Lacticaseibacillus rhamnosus* GG	0.96 ± 0.04 *b	5.35 ± 0.85 a	49.3 ± 2.0 *a	47.3 ± 2.1 a	22.1 ± 0.3 *b	82.0 ± 1.1 a	223.0 ± 11.1 a	57.7 ± 2.2 b	0.25 ± 0.05 *b	0.97 ± 0.09 a
*Lacticaseibacillus rhamnosus* 8/4	0.99 ± 0.04 *b	5.60 ± 0.58 a	74.1 ± 3.7 *a	61.4 ± 3.8 *b	26.2 ± 0.6 b	97.0 ± 6.1 a	283.0 ± 9.4 *a	63.4 ± 0.2 *b	0.38 ± 0.02 b	0.98 ± 0.03 a
*Lacticaseibacillus rhamnosus* K	1.02 ± 0.02 b	5.65 ± 0.29 a	52.5 ± 2.9 a	56.5 ± 4.1 *a	23.2 ± 0.2 *b	91.1 ± 6.2 a	225.8 ± 11.2 a	62.1 ± 0.8 b	0.25 ± 0.03 *b	0.94 ± 0.05 a
*Ligilactobacillus salivarius* AWH	0.95 ± 0.00 *b	5.43 ± 0.17 a	67.8 ± 1.0 *a	51.3 ± 3.8 b	28.7 ± 1.0 *b	84.1 ± 6.4 a	236.7 ± 7.9 a	61.0 ± 0.6 b	0.50 ± 0.01 *b	0.95 ± 0.01 a

Data are expressed as mean ± standard deviation (*n* = 3). Means in each column followed by an upper star are significantly different (*p* < 0.05) based on Student’s *t*-test (fermented samples compared to the control sample). Means in each row followed by a letter are significantly different (*p* < 0.05) based on one-way ANOVA. FIC is expressed in fluorescence units per mg of d.m. The browning index is expressed in absorbance units (AU).

## Data Availability

Data sharing is not applicable.
